# Computer-Aided Diagnosis Evaluation of the Correlation Between Magnetic Resonance Imaging With Molecular Subtypes in Breast Cancer

**DOI:** 10.3389/fonc.2021.693339

**Published:** 2021-06-23

**Authors:** Wei Meng, Yunfeng Sun, Haibin Qian, Xiaodan Chen, Qiujie Yu, Nanding Abiyasi, Shaolei Yan, Haiyong Peng, Hongxia Zhang, Xiushi Zhang

**Affiliations:** ^1^ Department of Radiology, Third Affiliated Hospital of Harbin Medical University, Harbin, China; ^2^ School of Computer Science and Technology, Harbin Institute of Technology, Harbin, China; ^3^ Department of Pathology, Third Affiliated Hospital of Harbin Medical University, Harbin, China

**Keywords:** breast cancer, molecular subtypes, magnetic resonance imaging, computer-aided diagnosis, gradient tree boosting

## Abstract

**Background:**

There is a demand for additional alternative methods that can allow the differentiation of the breast tumor into molecular subtypes precisely and conveniently.

**Purpose:**

The present study aimed to determine suitable optimal classifiers and investigate the general applicability of computer-aided diagnosis (CAD) to associate between the breast cancer molecular subtype and the extracted MR imaging features.

**Methods:**

We analyzed a total of 264 patients (mean age: 47.9 ± 9.7 years; range: 19–81 years) with 264 masses (mean size: 28.6 ± 15.86 mm; range: 5–91 mm) using a Unet model and Gradient Tree Boosting for segmentation and classification.

**Results:**

The tumors were segmented clearly by the Unet model automatically. All the extracted features which including the shape features,the texture features of the tumors and the clinical features were input into the classifiers for classification, and the results showed that the GTB classifier is superior to other classifiers, which achieved F1-Score 0.72, AUC 0.81 and score 0.71. Analyzed the different features combinations, we founded that the texture features associated with the clinical features are the optimal features to different the breast cancer subtypes.

**Conclusion:**

CAD is feasible to differentiate the breast cancer subtypes, automatical segmentation were feasible by Unet model and the extracted texture features from breast MR imaging with the clinical features can be used to help differentiating the molecular subtype. Moreover, in the clinical features, BPE and age characteristics have the best potential for subtype.

## Introduction

Breast cancer is the most common cancer in females, and it is a heterogeneous disease with different subtypes, varying clinical presentations, and treatment responses (1, 2). In breast cancer, gene expression profiling has revealed four main intrinsic molecular subtypes that show apparent differences in the gene expression patterns: luminal A, luminal B, triple-negative, and human epidermal growth factor receptor 2(HER2)-enriched. The intrinsic molecular subtypes have different treatment responses, prognosis, phenotypic presentations, recurrence-free, and disease-specific survival, leading to molecular subtype-based recommendations for systemic therapy (3–5). The molecular subtypes follow either gene expression profiling or immunohistochemical (IHC) surrogates from invasive tissue sampling. There are some limitations to the methods. First, needle biopsy is often used for the preoperative diagnosis. It may capture only a snapshot of the tumor tissue that may be subject to the selection bias and may not be entirely representative of the epigenetic, genetic, phenotypic alterations of the entire tumor. Second, the tumor tissue may have changed over time due to the treatment, i.e., it may change from a stem-like, a differentiated drug-sensitive phenotype, a therapy-resistant to epithelial-mesenchymal transition. Besides, there is a strong argument for the alternative of tumor features during the treatment, i.e., receptor status and molecular subtypes may have changed during the tumor treatment. Therefore, there is a demand for additional alternative methods that can allow the differentiation of the breast tumor into molecular subtypes precisely and conveniently.

Magnetic resonance imaging (MRI) is increasingly being used for breast cancer because it has higher sensitivity than ultrasonography and mammography (6–8). Many imaging tools based on computer-aided diagnosis (CAD) technologies have been developed with computer applications development to enhance diagnostic accuracy. CAD also has the potential to improve observer reproducibility in dynamic contrast material-enhanced MR imaging in differentiating benign from malignant lesions (9–11). If breast molecular subtypes could be identified from the MR image, it would be a valuable additional diagnostic tool. It would provide complementary information to the diagnosis of immunohistochemical surrogates while bypassing the need for costly and difficult molecular subtyping. Some pilot studies (12–14) showed the relationship between breast cancer molecular subtyping and MR imaging features correlated with different breast cancer molecular subtypes, but the generalization of these results is limited due to the utilization of different MRI protocol scanners.

The purpose of the present study was to determine suitable optimal classifiers and investigate the general applicability of CAD to associate between the breast cancer molecular subtype and the extracted MR imaging features.

## Materials and Methods

### Ethics and Consent

The study was a retrospective study, and the institutional ethics committee approved the protocol of our university for human research. Informed consent was obtained from all the patients.

### Breast MR Imaging Data Sets

Breast MR imaging studies were selected from the Picture Archiving and Communication Systems (PACS), which links clinical information with radiological and pathological reports to MR images. From April 2015 to December 2018, a total of 269 patients were included in our study. Five patients were excluded from the study group because the pathological results were lacking or imprecise. The final study group therefore consisted of 264 patients (mean age: 47.9 ± 9.7 years; range: 19–81 years) with 264 breast cancers (mean size: 28.6 ± 15.86 mm; range: 5–91 mm) who underwent core-needle biopsy or surgery were included in our study.

### MRI Acquisition Protocol

MR images were obtained using a 3.0T MR scanner (Philips Achieva 3.0T). The patients adopted a prone position and put their breasts into the dedicated phased-array breast coil. Imaging parameters for DCE-MRI were are as follows:

Axial T1-weighted imaging (repetition time (TR) = 495 ms; echo time (TE) = 10 ms; slice thickness/gap = 3 mm/0 mm; matrix = 512; number of signal averaged (NSA) = 1; field of view (FOV) = 340 mm × 340 mm); axial T2-weighted imaging (TR = 4213 ms, TE = 120 ms, slice thickness/gap = 3 mm/0 mm, matrix = 512, NSA = 1, FOV = 340 mm × 340 mm); T2-weighted fat-saturated imaging using a spectral selection attenuated inversion recovery (SPAIR) (TR = 4216 ms, TE = 60 ms, inversion delay (IR) = 120 ms, slice thickness/gap = 3 mm/0 mm, matrix = 352, NSA = 1, FOV = 340 mm × 340 mm); and T1-weighted high-resolution isotropic volume examination (THRIVE) (TR = 4.4 ms, TE = 2.2 ms, flip angle = 12°; matrix = 352; FOV = 340 mm × 340 mm; number of sections = 110; acquisition time: 256 s). MR imaging data sets were acquired once before gadolinium (Gd)-diethylenetriamine penta-acetic acid (DTPA) (Bayer Scheming Pharma AG, Berlin, Germany) injection and at 90-s intervals upon injection of 0.1 mmol/kg Gd-DTPA (followed by an intravenous saline flush of 20 ml), for a total imaging duration of 5 to 8 min.

### Tumor Segmentation

We chose the first sequence of DCE-MRI for segmentation and features extraction. The contrast of the image was enhanced by normalizing the histogram of the original image.

Unet model was applied to the segmentation part of the breast tumors because it is a network structure widely used in the field of medical image segmentation. Unet is a fully convolutional neural network, which can combine low-level information with high-level information at the same time. The low-level information retains the spatial features, while the high-level information extracts the in-depth abstract features. The model consists of two parts, namely the encoder and the decoder. The encoder is composed of a convolution layer and a down-sampling layer to extract in-depth abstract features. The decoder part consists of a convolutional layer and deconvolution layer, which upsamples in-depth features to the original image’s size. The network structure of Unet is as follows, the down-sample layer is the red arrow in the figure, which is realized by max-pooling and the up-sample layer is the green arrow in the figure, which is realized by deconvolution. Skip connection is represented by a gray arrow, which combines low-level features and high-level semantic features to realize up-sampling step by step. Finally, the feature map is converted into the probability graph through softmax operation.

### Tumor Feature Extraction and Selection

Features were extracted from the generated images which only contained tumor regions, including shape features, texture features and clinical features.

A series of quadratic statistical features could be calculated based on the normalized Gray-Gradient Co-occurrence Matrix (GGCM). Based on the normalized gray gradient co-occurrence matrix (GGCM), a series of quadratic statistical features can be calculated. In this experiment, the GLCM was used to extract the 48 grayscale features (entropy, homogeneity, correlation, and energy with the step of 1, 2, and 3, respectively, and the direction of 0, 45, 90, and 135, respectively). Clinical features were extracted including whether the patient was menopausal, TIC curve type, BPE classification type, patient age and tumor length. The 13 shape features were composed of roundness, aspect ratio, average normalized radial length, normalized radial length standard deviation, average normalized entropy of radial length, area ratio, boundary roughness, length-width ratio, lobular number, degree of needling, direction angle, normalized circumference, and normalized contour.

We extracted the features of the images, including the shape features, the tumors’ texture features (**Figure 3**), and the clinical features. All 51 images of luminal A were divided into five dissecting subsets, and the luminal B, TN and Her2 data set were also divided into five subsets. Each time, take one of the luminal A, luminal B, Her2, and TN subsets as the test sets and the other four subsets of luminal A, luminal B, TN, and Her2 training sets. We were then training the model or hypothesis function according to the training sets. Put this model on the test set and get the classification rate. Finally, we calculated the average classification rate five times as the model’s real classification rate or hypothesis function.

### Tumor Classification

Different tumor subtypes (Luminal A, Luminal B, HER2-enriched, TN) were tested using the extracted features. The extracted features were input into the Gradient Tree Boosting (GTB) classifier for experiments, and the results compared with Random Forest (RF), Support Vector Machine (SVM), Logistic Regression (LR), and Decision Tree (DT) classifiers.

The algorithm’s core of gradient boosting is that each tree learns from all previous trees’ residuals. The negative gradient value of the loss function in the current model was used.

$$r_{mi}=-{\left[\frac{\partial L(y_{i},f(x_{i}))}{\partial f(x_{i})}\right]}_{f(x)=f_{m-1}(x)}$$

As an approximation of the residual in the lifting tree algorithm, a classification tree is fitted. Gradient lift is one of the Boost algorithms, or an improvement on the original Boost algorithm, which assigns equal weight to each sample at the beginning of the algorithm, meaning that everyone is equally important at the beginning. In every training model, we will make an estimate of the data points, so at the end of each step, we need to deal with the weight value. Moreover, the means of processing is by increasing the wrong classification points’ weight and simultaneously reducing the correct classification point. That is to say, if some points are always wrong, then they will be “serious concern” and are assigned a very high weight. After N iterations (20 in this paper), there will be an N simple base classifier (basic learner). Finally, we put them together, and they can be weighted (error rate, the greater the base classifier, the smaller the weight value, the smaller the error rate of the base classifier weight value is larger), or vote for a final model.

This Gradient Boost is quite different from a traditional Boost in that it is calculated to reduce the last residual and reduce this residual, and a new model can be built in the direction of the Gradient reduction. In Gradient Boost, each new model was built to reduce the residual from the previous model in the gradient direction, and significantly different from the traditional Boost algorithm that weights the correct and incorrect samples.

### Evaluation Index

Three evaluation indexes, Accuracy (ACC), F1-score, and SCORE, were used in the experiment.

Precision refers to the percentage of pixels whose predicted result is an upbeat class, and the actual result is a positive class. The higher the precision value is, the higher the model segmentation results to the calibration results. The formula is as Eq.5. The higher the value of precision is, the better the performance of the model is.

(1) $$\text{Precision}=\frac{TP}{TP+FP}$$

F1-score combines the result of precision and TPR, and the formula is as Eq.6. The higher the value of F1-score is, the better the performance of model is.

(2) $$F1-score=2*\frac{precision*TPR}{precision+TPR}$$

The closer the score is to 1, the better the performance of the classifier is.

### Pathological Diagnoses

All breast lesions were confirmed histologically via surgery or biopsy. Lesions were divided into subgroups, as described in **Table 1**. A pathologist made all diagnoses with many years of experience in pathological breast examination.

**Table 1:** Baseline Characteristics.

Characteristic	All patients (n = 264)	Luminal A (n = 51)	Luminal B (n = 124)	HER-2 (n = 46)	TN (n = 43)
Age (y)*	47.9±9.70 (19-81)	48.0±9.23 (24-81)	47.2±9.98 (19-71)	49.7±8.26 (37-69)	48.9±10.69 (23-70)
Tumor diameter (mm)*	28.6±15.86 (5-84)	22.6±13.16 (5-68)	27.9±15.47 (4-84)	34.4±17.98 (11-91)	32.2±14.68 (5-62)
Menopausal status					
Premenopausal	151	32	77	20	22
Postmenopausal	113	19	47	26	21
TIC					
1	12	2	9	0	1
2	104	16	50	15	23
3	148	33	65	31	19
BPE					
1	97	18	53	13	13
2	104	27	43	20	14
3	54	5	24	11	14
4	9	1	4	2	2

*Data are means, with ranges in parentheses.

### Statistical Analysis

This study is interested in the association of imaging features and clinicopathological features with different molecular subtypes. The features were extracted from GGCM, and the classifier’s performance was compared in terms of F1-score, ACC, and score. The predictive performance combined with imaging features extracting from optimal classifier and clinical features was also evaluated with F1-score, ACC, and GTB classifier scores. For the classification of the four molecular subtypes, a classification matrix and the ROC curve using a one-vs-all approach were generated. The area under the curve (AUC), accuracy, sensitivity, and specificity were calculated. All data were analyzed using version 19.0 SPSS software (IBM Corp., Armonk, NY, USA).

## Results

The patient demographic and cancer features are shown in **Table 1**. There were 51 luminal A (19.3%), 124 luminal B (47%), 43 triple-negative (16.3%), and 46 HER2-enriched (17.4%) in the 264 breast cancers (mean size: 28.6 ± 15.86 mm; range: 5–91 mm).

**Figure 1:**
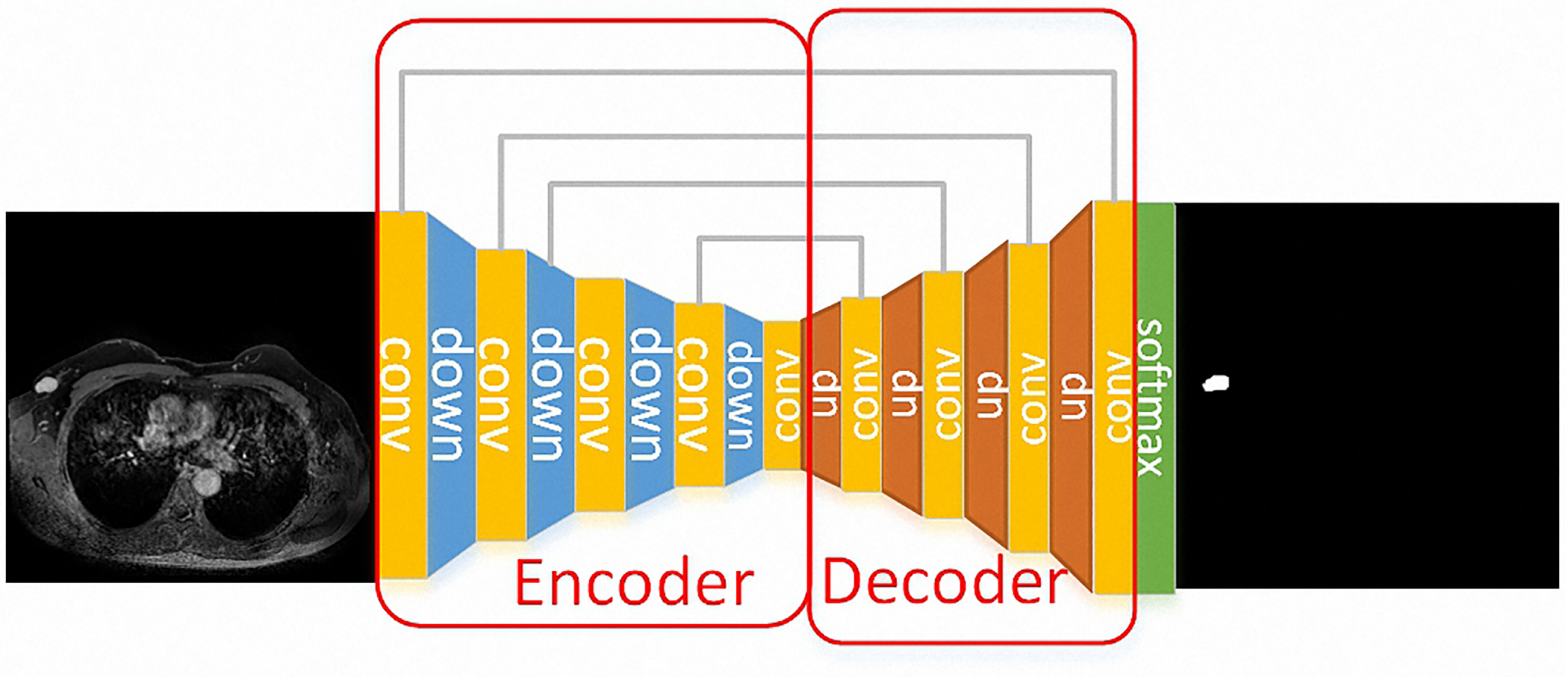
A case for the segmentation process. The Unet model was used for the segmentation of the breast tumors. The down-sample layer is the blue module in the figure, which is realized by max-pooling. The up-sample layer is the red module in the figure, which is realized by deconvolution. Moreover, skip connection is represented by a gray line, which combines low-level features and high-level semantic features to realize up-sampling step by step. Finally, the feature map is converted into the probability graph through softmax operation.

**Figure 2:**
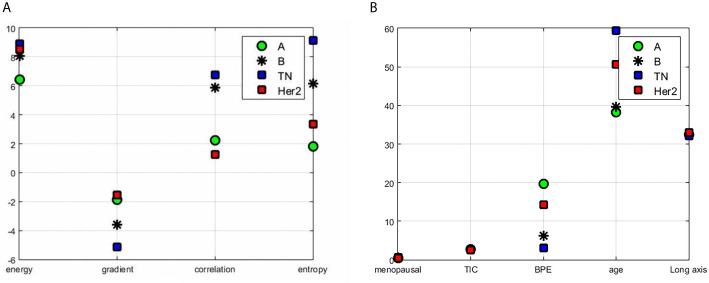
Results of features contrast of the different subtypes: **(A)** the representative gradient features including energy, gradient, correlation, and entropy; **(B)** the contrast results for clinic features which contain menopausal, TIC curve type, BPE classification value, patient age, and tumor length among the four types.

**Figure 3:**
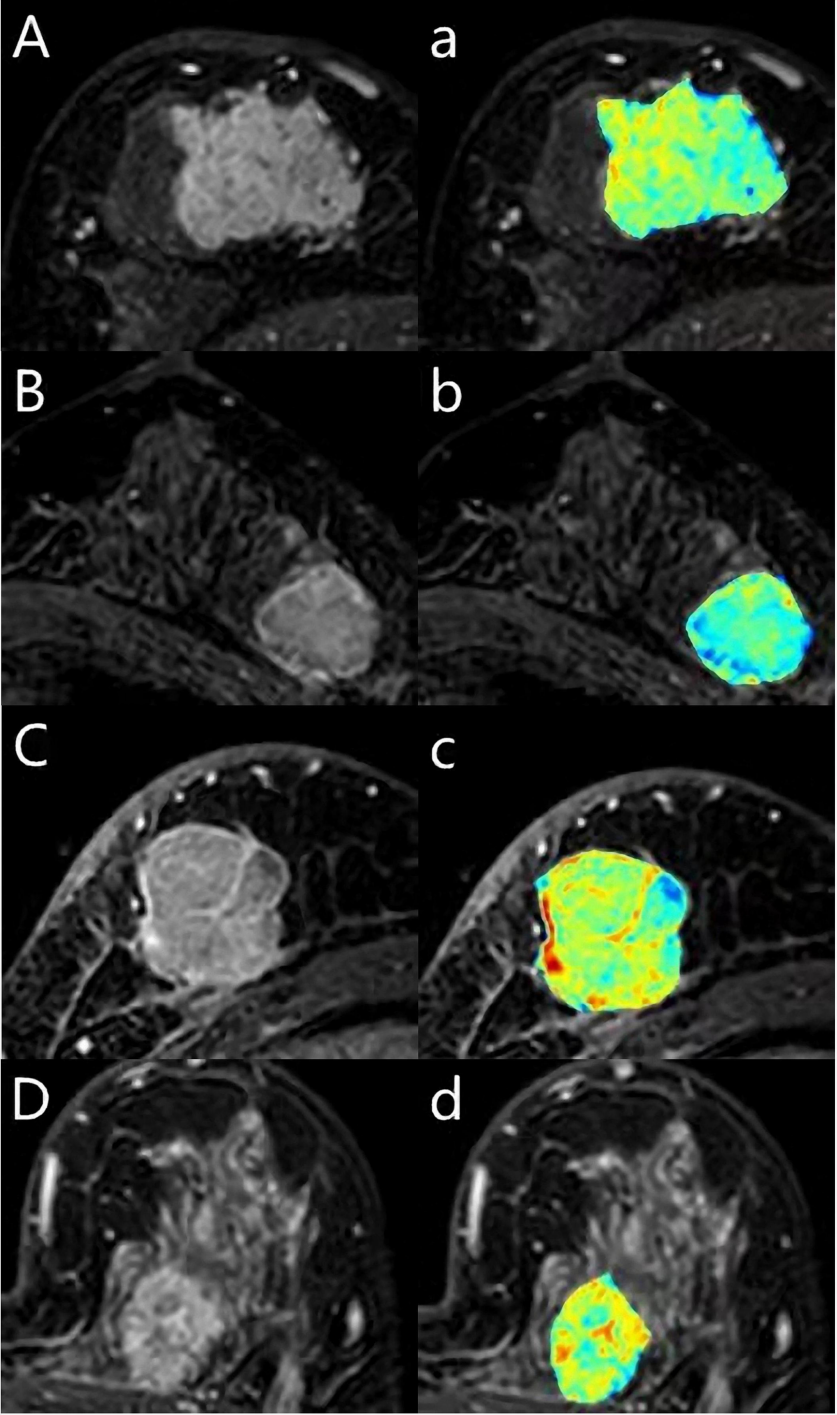
A 62-year-old woman with Luminal A breast cancer **(A, a)**, a 55-year-old woman with Luminal B breast cancer **(B, b)**, a 59-year-old woman with triple-negative breast cancer **(C, c)**, a 43-year-old woman with human epidermal growth factor receptor 2 (HER2) breast cancer **(D, d)**. The first DCE sequence (ABCD) and the texture map with colors (abcd) were shown.

The segmentation process and results obtained by preprocessing were compared with those obtained without preprocessing and are shown in **Figure 1**. The tumors were segmented clearly by the Unet model automatically. The representative gradient features, including energy, gradient, correlation, and entropy, are shown in **Figure 2A**. The contrast results for clinic features among the four types are shown in **Figure 2B**. We extracted the images’ features, including the shape features, the tumors’ texture features (**Figure 3**), and the clinical features. All the extracted features were input into the GTB classifier for experiments, and the results compared with the RF, SVM, LR, and DT classifiers, and the results are shown in **Table 2**. The results show that the GTB classifier is superior to other classifiers, which achieved F1-Score 0.72, ACC 0.81, and score 0.71.

**Table 2:** The classification results of the five classifiers.

Method\Result	F1-Score	ACC	score
GTB	0.72	0.81	0.71
RF	0.51	0.67	0.51
SVM	0.54	0.69	0.64
LR	0.43	0.64	0.44
DT	0.45	0.65	0.45

**Figure 4:**
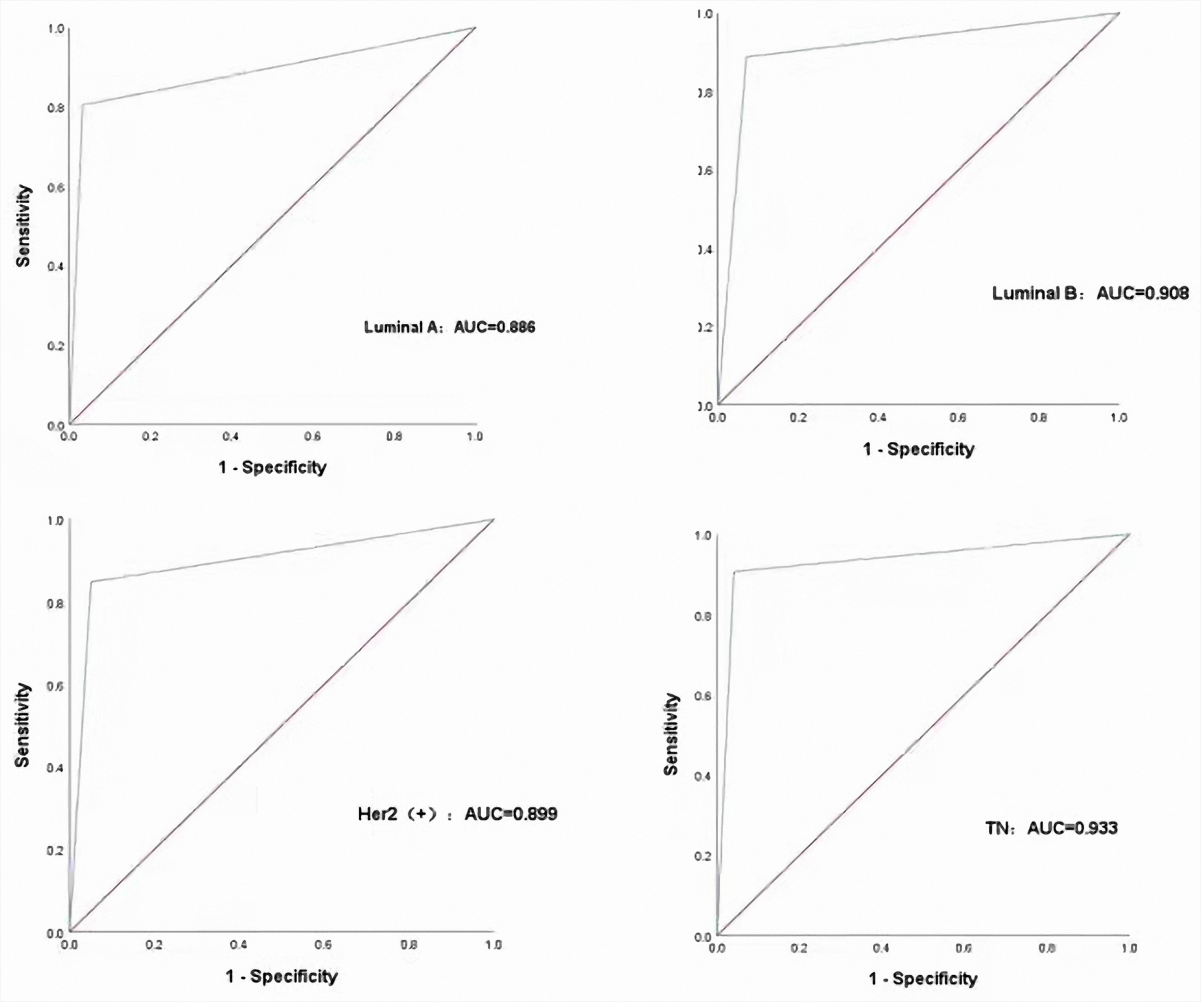
Performance of the CAD in classifying different molecular subtypes with the four subtypes.

Then, we input the extracted features into the GTB classifier according to different combinations and finally found that the features associated with the clinical features are the optimal features to different breast cancer subtypes, and the results are shown in **Table 3**. Molecular subtypes can be predicted with the GTB classifier. From the results of the classification (**Figure 4**), the TN subtype reached the highest AUC of 0.933, while the AUC of Luminal B reached 0.908, the AUC of Her-2 reached 0.899, the AUC of Luminal A 0.886. The sensitivity of Luminal A, Luminal B, Her-2, TN are 80.4%, 88.7%, 84.8%, 90.7%, while the specificity are 93.5%, 90.9%, 95.8%, 93.1% respectively.

**Table 3:** The results of ablation studies.

Feature\Result	F1-Score	ACC	score
Texture+clinical features	0.82	0.87	0.81
Clinical features	0.69	0.75	0.68
Shape+ clinical features	0.67	0.78	0.67
Texture+shape	0.43	0.63	0.44
Shape+texture+ clinical features	0.72	0.81	0.71
Texture+shape+BPE	0.69	0.79	0.68
Texture+shape+BPE+long axis	0.59	0.73	0.59
Texture+shape+BPE+long axis+age	0.70	0.80	0.69
Texture+shape+BPE+long axis+age+TIC	0.69	0.78	0.68

## Discussion

The current model approach of replacing molecular subtyping with computer extracted imaging features is continually being developed and validated, a technique that can provide the best prognostic benefit to patients without adding additional cost or delaying treatment planning. Many studies (15–17) have led to very considerable advances in detecting breast cancer molecular subtypes. Nevertheless, the prediction accuracy of most studies, as well as the reproducibility of the model, still needs further investigation.

Although this is a preliminary study, we showed that computer-assisted extraction of image features could be used to help identify the breast cancer molecular subtypes. In this work, we used Unet model and GTB for segmentation and classification. One of our methodology’s key benefits was automatically segmented and extracted features of the tumors. The Unet model is a fully convolutional neural network, which can combine the low-level information with the high-level information at the same time. It has shown promising results in many different applications. However, there have been few studies in breast tumor segmentation (18, 19). After segmentation, the tumors’ morphological features, such as the shape and the margins, were shown more clearly. Our experiment employed GGCM and GLCM methods to extract 51 grayscale features and 15 gradient features, and we collected the clinical features, which contained whether patients were menopausal, TIC curve types, BPE grade types, patient age, and tumor length. The grayscale features, gradient features, and shape features of the tumor were extracted and input into the GTB classifier to classify breast cancer’s four molecular subtypes. We observed that the F1-Score, ACC, and GTB classifier score was superior to other classifiers from the classification results. From **Table 3**, we found that the combination of texture features with clinical features had the best performance for predicting genotyping with an ACC value of 0.87, whereas the combination of texture features with shape predicted the worst genotyping effect with an ACC value of only 0.63. The results indicate that clinical features are crucial for the genotyping of tumors. It is not essential for subtyping of the tumor to add the shape features. Our result is so different from the other studies. Leithner (20) extracted radiomic features to assess breast cancer receptor status and molecular subtype’s diagnostic value. Radiomics analysis of manually segmented tumors was from the initial DCE-MRI and apparent diffusion coefficient (ADC) maps. They used a multi-layer perceptron feed-forward artificial neural network (MLP-ANN) for separation, and the ACC was 0.86 for the separation of TN from the other subtypes. However, their study used only the imaging parameters, not adding the clinical features. Maciej A (21) extracted 23 imaging features from breast tumors from MR imagings. The features contained morphologic, textural, and dynamic features but not any clinical features. They found that the luminal B subtype of breast cancer is associated with MR imaging features related to the tumor’s enhancement dynamics.

From classification results of texture features combined with shape features and results of texture features, shape features, and clinical features, we can see that ACC was increased by 18% with the help of clinical features. In order to determine the significance of clinical features for subtyping, we conducted experiments with different clinical features. Furthermore, we can conclude that BPE and age features have the best effects for genotyping. By adding the BPE features, ACC was increased by 16%, and by adding the age features, ACC increased by 7%.

CAD may be a valuable complementary method to differentiate the breast cancer molecular subtypes. Our work showed that the tumors can be segmented automatically by the Unet model and the combination of the texture features especially BPE and age features had the best performance for predicting genotyping. We found that TN subtype reached the highest AUC of 0.933 with GTB. Such finding may indicate that TN breast cancer was more heterogeneous compared with other subtypes. One of the possible explanations for the findings may be that the TN subtypes demonstrated more necrosis, so the texture may be more features in the images. That results were consistent with some studies (22, 23).

Our preliminary study had some limitations. First, our images were obtained from a single site. The sample size of 264 tumors and the different subtypes were numerically unbalanced; almost half of the cases were Luminal B. However, although the sample size was not significant and balanced, we discovered the association between the subtypes of breast cancers and the MR imagings. Moreover, additional studies with a more excellent sample of breast cancers are required to establish the clinical value of CAD in the subtypes’ differential diagnosis. Second, no formal training for the processed images was used in our study. Although the processed images’ features were familiar to the radiologists, a training set to allow radiologists to become familiar with the CAD method might enhance their confidence to use it.

## Conclusions

Our clinical investigation of 264 breast lesions showed that automatical segmentation were feasible by Unet model and the extracted texture features from breast MR imaging with the clinical features can be used to help differentiating the molecular subtype. Moreover, in the clinical features, BPE and age features have the best potential for subtype. The ability of CAD to identify breast cancer molecular subtype has enormous potential clinical benefits, so further large prospective studies are required to fully determine the potential role of CAD.

## Data Availability Statement

The data sets presented in this study can be found in online repositories. The names of the repository/repositories and accession number(s) can be found in the article/supplementary material.

## Ethics Statement

All procedures performed in this study involving human participates were in accordance with the ethical standards of the institutional and/or national research committee and with the 1964 Helsinki declaration and tis later amendments or comparable ethical standards. Written informed consent to participate in this study was provided by the participants’ legal guardian/next of kin. Written informed consent was obtained from the individual(s) and minor(s)’ legal guardian/next of kin, for the publication of any potentially identifiable images or data included in this article.

## Author Contributions

WM, YS, and NA created the data sets, interpreted the data, and defined the clinical labels. XC, SY, and HP developed the network architecture and training and testing setup. WM, HQ, and QY created the figures and performed statistical analysis. WM wrote the manuscript. WM, HZ, and XZ provided the clinical expertise and guidance on the study design. HZ and XZ supervised the project. All authors contributed to the article and approved the submitted version.

## Funding

The work was supported by the National Natural Science Foundation of China (81901701), the Postdoctoral Science Foundation of Ministry of Heilongjiang Province (LBH-Q20123), and Harbin Medical University Cancer Hospital Haiyan fund (JJZD2019-1, JJMS2021-31).

## Conflict of Interest

The authors declare that the research was conducted in the absence of any commercial or financial relationships that could be construed as a potential conflict of interest.
